# Clinical Impact of the Temporal Relationship between Depression and Type 2 Diabetes: The Fremantle Diabetes Study Phase II

**DOI:** 10.1371/journal.pone.0081254

**Published:** 2013-12-04

**Authors:** David G. Bruce, Wendy A. Davis, Violetta Cetrullo, Sergio E. Starkstein, Timothy M. E. Davis

**Affiliations:** 1 School of Medicine and Pharmacology, University of Western Australia, Crawley, Western Australia, Australia; 2 School of Psychiatry and Neuroscience, University of Western Australia, Crawley, Western Australia, Australia; University of Texas Health Science Center at San Antonio, United States of America

## Abstract

**Background:**

The clinical features of type 2 diabetes may differ depending on whether first depression episode precedes or follows the diagnosis of diabetes.

**Methods:**

Type 2 patients from the observational community-based Fremantle Diabetes Study Phase II underwent assessment of lifetime depression using the Brief Lifetime Depression Scale (developed and validated for this study) supplemented by information on current depression symptoms (Patient Health Questionnaire, 9-item version) and use of antidepressants. Patients were categorized as never depressed (Group 1), having had depression before diabetes diagnosis (Group 2), diagnosed with depression and diabetes within 2 years of each other (Group 3) and having depression after diabetes diagnosis (Group 4).

**Results:**

Of 1391 patients, 20.8% were assigned to Group 2, 6.0% to Group 3 and 14.5% to Group 4. In Group 2, depression occurred a median 15.6 years before diabetes onset at age 37.2±14.7 years. These patients had similar clinical characteristics to never depressed patients except for reduced self-care behaviours and having more symptomatic peripheral arterial disease. In Group 4, depression occurred a median 9.9 years after diabetes onset at age 59.8±13.0 years. These patients had long duration diabetes, poor glycaemic control, more intensive management and more diabetic complications. Group 4 patients had more current depression than Group 2 but were less likely to be receiving antidepressants.

**Conclusions/Interpretation:**

The clinical features of depression and type 2 diabetes are heterogeneous depending on their temporal relationship. There may be corresponding differences in the pathogenesis of depression in diabetes that have implications for diagnosis and management.

## Introduction

Both type 2 diabetes and depression contribute substantially to the global burden of disease. They coincide more often than expected [1]–[3] and the combination of conditions features worse glycaemic control, poorer quality of life and an increased risk of chronic complications and premature death than diabetes alone [4]–[7]. Their association is bi-directional with depressive disorders increasing the risk of incident type 2 diabetes [8], [9] and diabetes increasing the incidence of depression [1]. The former directional association may be stronger than the latter [10].

The mechanisms underlying these associations remain poorly understood. Depression is potentially diabetogenic because of lax self-care behaviours and associated physical inactivity and obesity [11], but chronic inflammation, changes in the hypothalamic-pituitary-adrenal axis and the effects of antidepressant medications could also contribute [12], [13]. When depression first occurs in patients with established diabetes, aetiological factors may include the stress and burden of managing a complex disease associated with chronic complications [2]. However, while type 2 diabetes is largely a disorder of aging with peak onset in later life, depression onset is most common earlier in life.

In view of these considerations, we hypothesized that there would be potentially important clinical differences in diabetes onset, management and outcomes depending on whether depression preceded diabetes or *vice versa*, e.g. diabetes onset or diabetic complications could occur at an earlier age in those with previous depression. Because of a paucity of published data relevant to this hypothesis, we assessed lifetime depression and age at first depressive episode in type 2 participants in the Fremantle Diabetes Study Phase II (FDSII) [14] and compared a range of demographic and clinical characteristics in patient groups classified by lifetime depression status, specifically whether depression preceded or followed diabetes diagnosis.

## Patients and Methods

Written informed consent was obtained from the participants of this study. The individual studies described below were approved by the Human Research Ethics Committee of the South Metropolitan Area Health Service of Western Australia.

The FDSII is an ongoing, observational study of known diabetes conducted in a postcode-defined geographical area surrounding the port city of Fremantle in the Australian state of Western Australia. The recruitment period for FDSII was between 2008 and 2011. Patients with diabetes were identified using available hospital, clinic and family practice patient lists, widespread local advertising (media, pharmacies, optometrists, etc) and mail-outs to registrants of the Australian National Diabetes Supply Scheme then invited to participate in a longitudinal study after confirmation of the diagnosis of diabetes. Details of these procedures as well as sample characteristics including classification of diabetes types and non-recruited patients have been published elsewhere [14], [15]. In FDSII, 4,727 diabetic patients were identified in the local population of ∼157,000 in 2008 (crude diabetes prevalence 3.0%) during the 3-year recruitment period of whom 1,732 (36.6%) were recruited. The current study is based on baseline data from the 1,551 participants (89.5%) with clinically-defined type 2 diabetes.

### Clinical assessment

Each participant underwent a baseline assessment that comprised a comprehensive questionnaire, physical examination and fasting biochemical tests [14]. Diabetes type was assessed from treatment history, body mass index (BMI), age at diagnosis, and nature of first presentation and/or self-identification. Non-insulin treated patients and those ≥60 years of age at diagnosis were usually considered to have type 2 diabetes, as were patients <60 years of age at diagnosis and taking insulin at the time of study entry but whose first treatment was not insulin. In these cases, case records were consulted for evidence of ketonaemia, as well as islet cell antibody, glutamic acid decarboxylase antibodies, serum insulin and C-peptide levels, if available. Ethnic background was based on self-selection, country/countries of birth and parents' birth, language(s) spoken at home and country of grandparents' birth. Details of all current medications were documented, including estimates of dates when long-term medications were first prescribed.

Current depression was assessed using the 9-item Patient Health Questionnaire (PHQ-9), a brief self-assessment of depressive symptoms that is valid and reliable with high sensitivity and specificity in both primary care and in community studies of diabetes [16]–[18]. Participants rate nine depressive symptoms and their frequency/persistence over the previous two weeks. The PHQ-9 was used to assess major and minor depression syndromes based on Diagnostic and Statistical for Mental Disorders (DSM-IV) criteria. Major depression requires the patient to have, for at least two weeks, five or more depressive symptoms present for more than half the days, with at least one symptom being either depressed mood or anhedonia. For minor depression, patients have to have two to four symptoms present for more than half of the days with one symptom being either depressed mood or anhedonia.

Complications were ascertained using standard criteria [14]. Micro- and macroalbuminuria were defined as a urinary albumin:creatinine ratio (ACR) ≥3.0 and ≥30.0 mg/mmol respectively in an early morning urine sample, neuropathy as a score of >2/8 on the Michigan Neuropathy Screening Instrument clinical portion [19] and retinopathy as any grade in either/both eyes on retinal photography using a non-mydriatic camera. The estimated glomerular filtration rate (eGFR) was calculated from the serum creatinine [20]. Peripheral arterial disease (PAD) was defined as an ankle:brachial index ≤0.90 or a diabetes-related lower extremity amputation [21]. In Phase I, prior hospitalizations accessed through the Western Australian Data Linkage System [22] provided important supplementary data for ascertainment of coronary heart disease and cerebrovascular disease but this source is not yet available for Phase II subjects and so these complications are based on self-assessment in the present study. Biochemical testing was carried out in the same nationally accredited diagnostic biochemistry laboratory.

### Assessment of lifetime depression

For FDSII, a novel questionnaire was devised by one of the investigators experienced in developing psychiatric instruments (SES), to assess lifetime prevalence of depression and age at time of first episode (see Table S1). This questionnaire, designated the Brief Lifetime Depression Scale (BLDS), was modelled on the PHQ-9 and included similar items, general format and language, but the participants were asked whether they had ever had a period during their lives lasting for two weeks or more when they experienced any of the following symptoms: “felt down, depressed or hopeless”, “had little interest in doing things”, and “were less able to enjoy things”. If they answered yes to at least one of these items, they were then asked if they had also experienced sleep difficulties, tiredness/little energy, appetite disturbance, feelings of failure, concentration difficulties, psychomotor symptoms and suicidal ideation at that time. Depression was classified using DSM-IV criteria and was considered to have been present if they had had at least 5 depression symptoms, one of which had been either depressed mood or anhedonia. The year in which the episode occurred was also recorded, along with its duration in weeks, months or years, whether the patient sought treatment, the type of treatment type prescribed, whether they had experienced multiple episodes, and the age at which the symptoms began.

The BLDS was validated in a convenience sample of 27 FDSII participants, including enrichment with those suffering current depression. Each underwent an additional assessment using the Structured Clinical Interview for DSM-IV (SCID) Affective Disorders Module administered by an experienced psychiatrist (SES) who was blind to the BLDS and PHQ-9 responses. Using SCID diagnosis as the gold standard, the BLDS criteria had 92.5% sensitivity and 100% specificity; all but one patient with a diagnosis of lifetime depression based on the SCID assessment also had depression on the BLDS. Another 21 patients completed the BLDS a second time one week after the first with a Kappa statistic of 0.72 for diagnosing lifetime depression and intraclass correlation coefficient of 0.80 for the total score.

### Patient categorisation

Participants were classified into four groups based on the relative timing of diabetes and first depression onset defined using BLDS, supplemented as described below by the presence/absence of current depression and antidepressant medication history. Group 1 were those who had never experienced depression. Group 2 comprised participants diagnosed with depression >2 years before diabetes onset. Group 3 were diagnosed with both depression and diabetes within 2 years to allow for potential uncertainty surrounding the timing of diagnosis. Group 4 comprised participants diagnosed with depression >2 years after diabetes diagnosis. Patients who screened negative on the BLDS but positive on the PHQ-9 were considered to have new-onset depression.

There were 71 FDSII participants taking antidepressant medications who screened negative for both lifetime and PHQ-9 depression. Two experts (SES, DGB) independently assessed whether the antidepressant medication was prescribed for depression or other reasons with consensus when there was initial disagreement. Forty-one were considered to be have been prescribed treatment for depression and were assigned to groups based on the date of first prescription (20 to Group 2, 6 to Group 3 and 15 to Group 4) while 23 were considered to have been treated for other conditions (anxiety, chronic pain, insomnia and urinary incontinence management) and seven were excluded with potentially unreliable responses (unclassifiable).

### Statistical analysis

The computer package IBM SPSS Statistics 19 (IBM Corporation, Armonk, New York, United States) was used for statistical analysis. Data are presented as proportions, mean±SD, geometric mean (SD range), or, in the case of variables which did not conform to a normal or log-normal distribution, median [inter-quartile range, IQR]. For independent samples, two-way comparisons for proportions were by Fisher's exact test, for normally distributed variables by Student's *t*-test, and for non-normally distributed variables by Mann-Whitney U-test. Multiple comparisons were by Fisher's exact or chi-squared tests for proportions, one-way ANOVA for normally-distributed variables, and the Kruskal-Wallis test for non-normally distributed variables. To further examine differences between groups 1 and 2, a nested case-control study was conducted with each group 2 case matched by age, sex and diabetes duration to a control from group 1 in a 1∶1 ratio. A two-tailed significance level of *P*<0.05 was used throughout.

## Results

### Sample characteristics and lifetime depression

Of the 1551 patients with type 2 diabetes (mean age 65.7±11.6 years, 51.9% male) recruited to the FDSII, 1443 (93%) completed the PHQ-9, 1399 (90%) completed the BLDS, and 1391 (90%) had sufficient data for classification. The 160 non-completers were the same age as the completers (66.0±14.6 years vs 65.6±11.2 years, *P* = 0.80) and had similar diabetes duration (10.0 [3.0–17.0] vs 8.8 [2.7–15.8] years, *P* = 0.27), but they were more likely to be female (57.5% vs 47.0%, *P* = 0.012), less educated (73.3% vs 88.0% beyond primary level, *P*<0.001), not fluent in English (18.21% vs 9.8%, *P* = 0.003), and to be Aboriginal (28.1% vs 4.2%) while being less likely to be of Anglo-Celt ethnicity (31.9% vs 55.9%, *P*<0.001).

After classification, 58.7% of the sample had no history of depression (Group 1), 20.8% had depression that preceded diabetes (Group 2), 6.0% developed depression within 2 years of diabetes diagnosis (Group 3) and 14.5% had depression that followed diabetes onset (Group 4; see Table 1). The prevalence of current depression in the sample was 5.7% for minor depression and 6.0% for major depression. Overall, 41.3% of the sample reported having had depression at some time i.e. the lifetime depression prevalence.

**Table 1:** 1,391 patients with type 2 diabetes grouped by depression status.

	Group 1	Group 2	Group 3	Group 4	*P*-value
	No depression	Depression before diabetes	Concomitant conditions	Depression after diabetes	
Number (%)	816 (58.7)	290 (20.8)	84 (6.0)	201 (14.5)	
Age at assessment (years)	67.1±10.9	62.6±10.6^b^	58.9±12.2^b,d^	67.0±11.5^d^	<0.001
Sex (% female)	40.3	54.1^b^	65.5^b^	56.2^b^	<0.001
Age at diabetes diagnosis (years)	56.9±11.7	55.6±10.6	53.7±12.9^a^	50.5±12.5^b,d^	<0.001
Diabetes duration (years)	8.7 [2.9–15.8]	5.0 [1.1–11.0]^b^	3.0 [1.0–6.8]^b^	15.8 [11.0–20.0]^b,d^	<0.001
Age at onset of depression (years)	-	37.2±14.7	52.8±12.6^d^	59.8±13.0^d^	<0.001
Years between diabetes and depression diagnosis	-	−15.6 [−25.8 to −7.4]	−0.2 [−1.1 to 0.6]	9.9 [4.7 to 15.6]	
Current depression (%)	0	19.3^b^	35.0^b,d^	41.1^b,d^	<0.001
Current major depression (%)	0	11.8^b^	18.8^b^	17.9^b^	<0.001
Current minor depression (%)	0	7.5^b^	16.2^b,^ c	23.2^b,d^	<0.001
On antidepressant medication (%)	2.8^e^	35.5^b^	23.8^b,^ c	30.3^b^	<0.001

a *P*<0.05, ^b^ *P*<0.01 compared with Group 1 as reference (unadjusted for multiple comparisons).

c *P*<0.05, ^d^ *P*<0.01 compared with Group 2 as reference (unadjusted for multiple comparisons).

e antidepressant use attributed to treatment for non-depression related conditions.

### Clinical features

Key diabetes and depression-related features of the four groups are summarised in Table 1. Group 2 patients, the largest group with a history of depression, reported their first depressive episode at mean age 37.2 years, a median 15.6 years before diabetes onset. In Group 4 patients, depression first occurred a median 9.9 years after diabetes diagnosis at mean age 59.8 years and they had developed diabetes at a younger age and had longer diabetes duration than the other groups. All depression groups had a female preponderance. Current (PHQ-9) depression was significantly more prevalent in Group 4 than Group 2 patients, explained by a significant excess of minor depression. Yet proportionally fewer of this group were taking antidepressants (Group 4 vs Group 2: 52.1% vs 75.7%, *P*<0.001). When considering patients taking antidepressants without current depressive symptoms (who could be considered to have treated or controlled depression), there were also significantly fewer of these in Group 4 than in Group 2 (31.6% vs 56.6%, *P* = 0.042).

Sociodemographic and clinical features of the four groups are summarised in Table 2. Patients in the three depression groups were less likely to be married or in a relationship, more likely to be current smokers and, in Group 4, less well educated and less likely to be fluent in English than those in reference Group 1. Group 4 patients were also receiving more intensive diabetes management, had worse glycaemic control and had more microvascular complications and more PAD and ischaemic heart disease than all other groups. In contrast, Group 2 patients had clinical features much more similar to never-depressed patients except for younger age, shorter duration diabetes and fewer microvascular complications when studied. To further explore possible differences between Groups 1 and 2, we conducted a nested case-control study and matched each Group 2 patient with a Group 1 patient by age, sex and diabetes duration. In this analysis (see Table 3), there were no differences between the groups in age at diabetes diagnosis, use of glucose-lowering treatments, glycaemic control or in most chronic complications, but Group 2 had higher smoking rates, more frequent claudication symptoms (although not more PAD) and fewer performed self monitoring of blood glucose.

**Table 2:** Demographic and clinical characteristics of the sample by lifetime depression status.

	Group 1	Group 2	Group 3	Group 4	*P*-value
	No depression	Depression before diabetes	Concomitant conditions	Depression after diabetes	
Education (% beyond primary level)	88.6	90.3	90.2	81.5a,d	0.029
Non fluent in English (%)	10.3	5.5^a^	10.7	13.4^d^	0.018
Married/partner (%)	68.1	58.6^b^	64.3	52.7^b^	<0.001
Alcohol use (standard drinks/day)	0.1 [0–1.5]	0.1 [0–0.8]	0.1 [0–1.5]	0.1 [0–1.2]	0.13
Current smoker (%)	6.4	12.8^b^	15.5^b^	16.4^b^	<0.001
BMI (kg/m^2^)	30.7±5.7	32.1±6.4^b^	32.6±6.4^b^	31.6±6.6	0.001
Fasting serum glucose (mmol/L)	7.1 [6.2–8.6]	7.0 [6.1–8.4]	7.1 [6.2–9.6]	7.7 [6.2–9.7]a,d	0.025
HbA_1c_ (%)	6.8 [6.2–7.5]	6.7 [6.1–7.5]	6.7 [6.0–7.9]	7.1 [6.5–8.1]^b,d^	<0.001
HbA_1c_ >7.0% (%)	39.1	40.3	36.9	52.0^b,^ c	0.009
Diabetes treatment (%):		a		^d^	<0.001
Diet	24.3	33.4	26.2	13.9	
Oral agents	54.8	51.4	56.0	47.3	
Insulin ± oral agents	21.0	15.2	17.9	38.9	
Systolic blood pressure (SBP; mmHg)	148±22	141±20^b^	145±22	149±25^d^	<0.001
Antihypertensive therapy (%)	75.7	70.7	58.3^b,^ c	80.6^c^	0.001
Total cholesterol (mmol/L)	4.3±1.1	4.4±1.1	4.7±1.1^b,^ c	4.4±1.1^b^	0.002
LDL-cholesterol (mmol/L)	2.3±0.9	2.4±0.9	2.6±1.0^b,^ c	2.4±0.9	0.004
Triglycerides (mmol/L)	1.5 (0.9–2.4)	1.6 (1.0–2.6)^a^	1.7 (0.9–3.1)^a^	1.6 (0.9–2.9)^b^	0.002
Lipid-lowering treatment (%)	69.6	66.6	53.6^b,^ c	74.6	0.005
Urinary albumin:creatinine (mg/mmol)	2.0 (0.1–46.7)	1.8 (0.1–26.8)	1.8 (0.1–33.7)	4.1 (0.6–26.0)^b,d^	0.011
eGFR<60 ml/min/1.73m^2^ (%)	18.1	11.9^a^	5.1^b,^ c	27.0^b,d^	<0.001
Retinopathy (%)	23.3	12.9^b^	11.4^a^	35.8^b,d^	<0.001
Peripheral neuropathy (%)	59.3	52.6	53.7	64.7^d^	0.040
Peripheral arterial disease (%)	22.4	18.3	15.9	28.9^d^	0.024
Claudication symptoms (%)	8.2	9.3	7.1	17.9^b,d^	0.001
Myocardial infarction/angina (%)	17.8	14.1	13.1	25.4a,d	0.010
Stroke (%)	4.0	5.2	3.6	6.0	0.58
Self-monitoring blood glucose (%)	88.2	79.2^b^	71.1^b^	84.3	<0.001

a *P*<0.05, ^b^ *P*<0.01 compared with Group 1 as reference (unadjusted for multiple comparisons).

c *P*<0.05, ^d^ *P*<0.01 compared with Group 2 as reference (unadjusted for multiple comparisons.

**Table 3:** Nested matched case control study comparing group 2 with group 1 patients matched by age, sex, and diabetes duration.

	Group 1	Group 2	*P*-value
	No depression	Depression before diabetes	
Number	290	290	
Matching variables			
Age (years)	62.4±10.2	62.6±10.6	0.83^a^
Male (%)	45.9	45.9	1.00^a^
Diabetes duration (years)	5.0 [1.9–11.3]	5.0 [1.1–11.0]	0.43^a^
Age at diagnosis of diabetes (years)	55.2±10.0	55.6±10.6	0.60^a^
Clinical variables			
Treatment (% diet/oral agents/insulin±orals)	32.1/53.4/14.5	33.4/51.4/15.2	0.97
Fasting serum glucose (mmol/L)	7.2 [6.0–8.7]	7.0 [6.1–8.4]	0.92
HbA_1c_ (%)	6.8 [6.1–7.5]	6.7 [6.1–7.5]	0.56
BMI (kg/m^2^)	31.4±6.1	32.1±6.4	0.18
Alcohol consumption (units/day)	0.1 [0–1.2]	0.1 [0–0.8]	0.75
Smoking status (% never/ex-/current)	50.5/43.6/5.9	39.1/48.1/12.8	0.005
Self-monitoring blood glucose (%)	89.9	79.2	<0.001
Myocardial infarction/angina (%)	13.1	14.1	0.80
Stroke (%)	2.4	5.2	0.10
Peripheral arterial disease (%)	16.2	18.3	0.57
Claudication symptoms (%)	3.4	9.3	0.003
Ln(urinary albumin:creatinine ratio)	1.7 (0.1–25.2)	1.8 (0.1–26.5)	0.77
Retinopathy (%)	14.3	12.5	0.30

a unpaired tests since these were matching variables; otherwise paired tests.

## Discussion

The main finding of the present study was that there were substantial clinical differences between patients with type 2 diabetes and a history of depression depending on whether depression pre-dated diabetes or first developed after the onset of diabetes. In Group 4, depression first occurred an average of a decade after the onset of the diabetes and was associated with poor glycaemic control despite intensive diabetes management and considerable morbidity from microvascular and macrovascular complications. All conventional chronic complications, except for stroke, were more prevalent in this patient group, and the associated disease and treatment burden was likely to be considerable. In Group 2, the first episode of depression was reported to have occurred a median 15 years before the onset of diabetes and there were few differences compared with never-depressed patients with no difference in diabetes onset and most chronic complications. Nevertheless, this patient group also had several adverse prognostic features that have been associated with depressive disorders [23], [24]. Fewer of them performed home blood glucose monitoring consistent with reduced treatment adherence [23] and more were current or ex-smokers consistent with the higher rate of symptomatic PAD [24].

We created an additional category, Group 3, in which both diabetes and depression developed at around the same, for methodological reasons. This group had clinical features that were intermediate between the other depression groups but comparisons are difficult given the small group size and differences in age and diabetes duration when they were studied. It is conceivable that psychological vulnerability explains why some patients become depressed at or soon after the diagnosis of diabetes and the planned longitudinal follow-up of the cohort should help clarify the clinical picture in this situation.

There were also between-group differences in relation to current depressive symptoms. Group 2 patients had less current depression than the other two groups. This may have been, in part, because of successful diagnosis and treatment. Proportionally more of this group were on antidepressants without reporting current depression symptoms, presumably indicating a prior response to initial and maintenance therapy. In addition, proportionally more of the patients in this group who had current depression symptoms were taking antidepressants, indicating that they had already been diagnosed. This is in contrast to Group 4 patients who had the highest prevalence of major and minor depression yet the lowest rate of antidepressant treatment. Mood disorders that first occur in the context of established diabetes appear to be more difficult to detect than at other times and these patients have a higher risk of remaining under-diagnosed and undertreated.

The strengths of the present study include the comprehensive nature of the clinical assessment conducted in a large community-based cohort and the use of the well-validated PHQ-9 depression assessment instrument. The limitations include the cross-sectional nature of the analysis, the reliance on patient self-reports for depression assessments including the use of a novel scale for assessing lifetime depression given the absence of a suitable, alternative instrument. The PHQ-9 has high negative predictive value but a positive predictive value of around 50% [17] indicating that a proportion of patients with current depressive symptoms may have been misclassified. This would not be expected to alter the main conclusions of the study appreciably given that group assignment was based largely on the BLDS. The PHQ-9 has been recommended as a diagnostic and management tool for collaborative care interventions in diabetic patients [25], in part because sub-syndromal depression may also have an adverse impact in diabetes [4], [5], [26]. Recall bias is expected to lead to an underestimate of lifetime depression [27] and would be expected to affect the results of this study by potentially misclassifying Group 2 cases into Groups 3 or 4. This would be expected to weaken the clinical differences that we have reported. The validation exercise indicated that the BLDS had equivalent diagnostic accuracy to a psychiatric diagnostic interview in our cohort and may be a useful tool in similar studies but the validation sample was small and the BLDS needs further validation.

There is limited information on the course of depression in diabetes and most existing prospective studies have been conducted in small, clinic-based samples and aimed to assess depression incidence and causes [10], [28]–[31]. A recent large telephone survey using the PHQ-9 reported that 20% of the sample had progressive depressive symptoms over 3 years, which appears to be consistent with our Group 4 findings [32]. A study of lifetime depression prevalence in a sample of women reported similar findings to our study in that the women with previous depression were also less likely to perform blood glucose monitoring [33]. Overall, 41% of our cohort reported having experienced depression during their lifetime. This lifetime estimate is considerably higher than reports from general population samples that had a 20% lifetime prevalence of mood disorders [34], [35] but appears consistent with the known strong bi-directional association with diabetes [1], [8].

The present findings have clinical implications for the diagnosis and management of depression in diabetes. Enquiring about lifetime depression histories appears worthwhile when type 2 diabetes is diagnosed. Patients with a prior history of depression would be expected to benefit from targeted programs aimed at preventing future depression episodes [36] and may require increased efforts aimed at improving self-care and reducing cigarette smoking, particularly as smoking is a risk factor for future depression and depression is a risk factor for starting and maintaining cigarette smoking [37]. Some patients develop depression at the time of diabetes onset and in others, the risk of depression appears to increase with time as hyperglycaemia becomes more difficult to control and complications develop. These data indicate that diabetic patients might benefit from repeated depression screening at specific intervals, such as at diagnosis, during therapy intensification and when complications develop. The association with micro- and macrovascular disease in this group suggests that some patients may have subclinical cerebrovascular disease that has been causally associated with late-onset depression [38].

In conclusion, there are substantial differences in the clinical features of both diabetes and depression depending on whether depression predates diabetes or first occurs in established diabetes. Differentiating these temporal patterns may assist future studies of the aetiology, diagnosis and management of depression in patients with type 2 diabetes.

## Supporting Information

Table S1 **Brief Life-time Depression Scale that was developed, validated and used to assess lifetime depression.**
(DOCX)
