# Supplementary material for: Clinical Impact of the Temporal Relationship between Depression and Type 2 Diabetes: The Fremantle Diabetes Study Phase II
Source: PLoS One. 2013 Dec 4;8(12):e81254. doi: 10.1371/journal.pone.0081254 (PMC3852722; doi:10.1371/journal.pone.0081254)
Supplement: Table S1 — Brief Life-time Depression Scale that was developed, validated and used to assess lifetime depression. (DOCX) [file pone.0081254.s001.docx]

Table S1: Brief Life-time Depression Scale that was developed, validated and used to assess lifetime depression.

Brief Lifetime Depression Scale

| *The following questions relate to whether you have had any of the following symptoms, at any time in your life, that lasted for more than two weeks…..* | | | | | | |
| --- | --- | --- | --- | --- | --- | --- |
| 1. **Did you have a period of time, lasting for more than two weeks, during which you had *any* of the following symptoms?** | | | | | **YES** | **NO** |
| a. | | Felt down, depressed, or hopeless? | | ❒ | | ❒ |
| b. | | Had little interest in doing things? | | ❒ | | ❒ |
| c. | | Were less able to enjoy things? | | ❒ | | ❒ |
| **If you answered *yes to any* of the above, please indicate if you experienced *any* of the following *at the same time:*** | | | | | **YES** | **NO** |
| d. | | had trouble falling or staying asleep, or sleeping too much? | | | ❒ | ❒ |
| e. | | felt tired or having little energy? | | | ❒ | ❒ |
| f. | | had poor appetite or over-ate? | | | ❒ | ❒ |
| g. | | felt bad about yourself – or that you were a failure or had let yourself or your family down? | | | ❒ | ❒ |
| h. | | had trouble concentrating on things, such as reading the newspaper or watching television? | | | ❒ | ❒ |
| i. | | moved or spoke so slowly that other people could have noticed? Or the opposite – were so fidgety or restless that you moved around a lot more than usual? | | | ❒ | ❒ |
| j. | | had thoughts that you would be better off dead or of hurting yourself in some way? | | | ❒ | ❒ |
|  | |  | | |  |  |
| 1. If **YES** to any of the above: | | | | |  |  |
|  | Have any of these episodes made it hard for you to do your work, take care of things at home, get along with other people, or involve yourself in your hobbies and pastimes? | | | | ❒ | ❒ |
|  |  | | | |  |  |
| 1. If **YES** to any of the above: | | | | |  |  |
| a. | | When was that? _______(year) | | |  |  |
| b. | | How long did it last for? ______ weeks OR _______ months OR ______ years | | |  |  |
| c. | | Did you seek treatment?  If YES, what type of treatment? ___________________________________ | | | ❒ | ❒ |
|  | |  | | |  |  |
| 1. Have you had more than one episode? | | | | | ❒ | ❒ |
|  |  | | |  | |  |
| 1. How old were you when the symptoms ***first*** began? | | |  | | | Age |
